# Genome-resolved insights into hydrocarbon-transforming and nitrate-reducing microbial communities from deep petroleum reservoir cores of the Nashpa Oil Field, Pakistan

**DOI:** 10.3389/fmicb.2026.1899441

**Published:** 2026-08-11

**Authors:** Sajida Tayyaba, Arshia Amin, Sobia Zulfiqar, Iftikhar Ahmed

**Affiliations:** 1 Department of Bioinformatics and Biosciences, Faculty of Health and Life Sciences, Capital University of Science and Technology, Islamabad, Pakistan; 2 National Culture Collection of Pakistan (NCCP), Land Resources Research Institute (LRRI), National Agricultural Research Centre (NARC), Islamabad, Pakistan

**Keywords:** hydrocarbon metabolism, metagenome-assembled genomes, microbial enhanced oil recovery, nitrate reduction, petroleum reservoir microbiome, shotgun metagenomics

## Abstract

**Introduction:**

Microbial communities from deep subsurface petroleum reservoirs are adapted to hydrocarbon-rich, oxygen-limited, and physicochemically extreme environments. However, genome-resolved knowledge of petroleum reservoir microbiomes from Pakistan remains largely unexplored.

**Methods:**

Shotgun metagenomic sequencing was used to investigate the functional and metabolic potential of microbial communities inhabiting deep subsurface petroleum reservoir cores from the Nashpa Oil Field, Pakistan, at depths of 3,770–4,315 m. Metagenome-assembled genomes (MAGs) were reconstructed and functionally annotated to assess taxonomic composition and predicted metabolic capabilities.

**Results:**

A total of 402 metagenome-assembled genomes (MAGs) were recovered, of which 216 were high-quality MAGs (≥90% completeness and ≤5% contamination). Taxonomic analysis showed dominance of *Pseudomonadota* and *Actinobacteriota,*, including genera such as *Alcanivorax, Marinobacter, Pseudomonas, Rhodococcus*, and *Thermohalobaculum*. Functional annotation revealed genes involved in hydrocarbon transformation, nitrate-linked respiration, oxygen-limited metabolism, oxidative phosphorylation, aromatic compound degradation, and cellular stress-response systems. However, markers of hydrocarbon degradation were detected only in a small subset of MAGs, suggesting taxon-specific metabolic specialization rather than broad community-wide enrichment. Genes associated with nitrate reduction and microaerophilic or anaerobic respiration suggested metabolic flexibility under the variable oxygen conditions typical of deep petroleum reservoirs. Stress-associated genes, including molecular chaperones and heat-shock proteins, further indicated putative adaptation to reservoir-associated environmental stress, although thermotolerance was not experimentally confirmed.

**Discussion:**

This study provides one of the first genome-resolved insights into deep petroleum reservoir microbiomes from Pakistan and identifies candidate microbial lineages carrying MEOR-relevant genomic traits for future functional validation.

## Introduction

Deep subsurface petroleum reservoirs represent extreme microbial habitats characterized by elevated pressure, limited oxygen availability, salinity, nutrient limitation, and hydrocarbon-rich conditions. Despite these harsh physicochemical conditions, diverse microbial communities can persist in reservoir environments and may contribute to hydrocarbon transformation, biogeochemical cycling, reservoir souring, gas production, and other subsurface ecological processes (Salam and Obayori, 2019; Magot et al., 2000). Understanding the predicted functional potential of these microbial communities is therefore important for interpreting deep biosphere ecology and for identifying microbial traits that may be relevant to reservoir management and microbial enhanced oil recovery (MEOR). Reservoir-associated microorganisms have been linked to several processes of potential relevance to MEOR, including biosurfactant production, biopolymer formation, biogas generation, and hydrocarbon transformation, all of which may influence oil mobilization and recovery efficiency under suitable reservoir conditions (Nam et al., 2023; Liu et al., 2025). However, these processes are strongly dependent on local reservoir conditions, including depth, temperature, salinity, pressure, nutrient availability, oxygen limitation, and hydrocarbon composition. Therefore, site-specific studies are required to understand how microbial metabolic potential varies among petroleum reservoir environments. Metagenomic approaches provide useful tools for investigating microbial communities in complex environmental systems. Shotgun metagenomic sequencing provides broader access to functional genes, metabolic pathways, and genome-level information (Pérez-Cobas et al., 2020; Durazzi et al., 2021). Advances in next-generation sequencing have enabled deeper profiling of microbial communities and have improved the ability to link microbial diversity with predicted ecological functions (Child et al., 2024; Fenibo et al., 2025). In particular, metagenomics can provide information on genes associated with hydrocarbon metabolism, stress response, energy conservation, and other reservoir-relevant processes (Zhang et al., 2021; Kumar et al., 2024). Shotgun metagenomics is especially valuable for recovering metagenome-assembled genomes (MAGs), which allow predicted functional traits to be linked with specific microbial lineages. MAG-based studies have expanded the current understanding of microbial diversity and metabolic potential in complex and poorly explored environments, including subsurface ecosystems (Vosloo et al., 2021; Mirete et al., 2025). Such genome-resolved analyses are useful for identifying uncultivated microbial lineages and candidate metabolic pathways; however, gene presence alone represents predicted functional potential and does not confirm gene expression, metabolic activity, or *in situ* performance (Wu et al., 2025). Therefore, functional interpretations from MAGs should be made cautiously and supported by a clear distinction between genomic potential and experimentally validated activity. Previous studies from petroleum reservoirs and hydrocarbon-impacted environments have reported microbial groups capable of surviving under hydrocarbon-rich and oxygen-limited conditions. These studies suggest that petroleum-associated microbial communities may contain genes involved in alkane activation, aromatic compound transformation, nitrate metabolism, oxygen-limited respiration, and cellular stress response. However, microbial functional potential can vary substantially among reservoirs because of differences in geochemical and environmental conditions. Therefore, findings from one reservoir cannot be directly generalized to another without site-specific investigation. Genome-resolved investigations are therefore required to identify reservoir-specific microbial lineages and their associated metabolic capabilities. Petroleum reservoirs harbors diverse microbial communities adapted to extreme conditions such as high temperature, pressure and salinity. These microbial communities include sulfate-reducing bacteria, methanogens, fermentative bacteria and nitrate-reducing bacteria that play important role in hydrocarbon transformation, reservoirs souring, corrosion and oil recovery process (Magot et al., 2000). Molecular studies have demonstrated that petroleum reservoirs contain complex microbial populations including thermophilic bacteria and archea that plays role in carbon and sulfur cycling under reservoirs conditions (Orphan et al., 2000). Microbial communities in petroleum reservoirs can have both detrimental and beneficial effects. Sulfate reduction may result in hydrogen sulfide production and reservoir souring whereas nitrate reduction can suppress sulfide formation and influence microbial community structure (Youssef et al., 2009). Previous studies have demonstrated that microbial population composition in petroleum reservoirs is strongly influenced by reservoirs temperature, salinity and pressure, leading to distinct ecological and metabolic adaptations among indigenous microorganisms (Shestakova et al., 2010).

In Pakistan, deep subsurface petroleum reservoir core microbiomes remain poorly characterized using genome-resolved shotgun metagenomics. In particular, limited information is available regarding the predicted functional traits of microbial communities from the Nashpa Oil Field. This knowledge gap restricts the understanding of the microbial ecology of Pakistani petroleum reservoirs and limits the identification of candidate microbial lineages carrying genes associated with hydrocarbon transformation, nitrate reduction, oxygen-limited metabolism, and stress adaptation. Although both sulfate-reducing and nitrate-reducing microorganisms are important components of petroleum reservoirs ecosystems, the present study specifically investigates nitrate-reducing microbial communities because of their ecological significance and potential role in mitigating reservoir souring. This study was designed as an exploratory genome-resolved survey of microbial functional potential in deep petroleum core samples from the Nashpa Oil Field. Shotgun metagenomics and MAG reconstruction linked functional genes to specific microbial lineages. The study aimed to identify high-quality MAGs, dominant reservoir-associated taxa, and genes related to hydrocarbon transformation, nitrate metabolism, oxygen-limited respiration, and stress response. All functional findings are interpreted as genomic potential, not confirmed metabolic activity or MEOR performance.

## Materials and methods

### Sample collection

Deep subsurface petroleum reservoir core samples were obtained from the Nashpa Oil Field, Haripur, Pakistan (33° 6′54.9684” N, 71° 5′43.9260″E), from depths ranging between 3,770 and 4,315 m. The samples comprised 20 reservoir core samples representing the Lockhart Formation (NW11-1 to NW11-10), Hangu Formation (NW11-11 to NW11-15), and Lumshiwal Formation (NW11-16 to NW11-20) (Table 1). Based on regional geothermal gradients reported for sedimentary basins of northern Pakistan, reservoirs temperature at these depths are expected to be approximately 95–140°C. However, direct formation temperature measurements for the samples intervals were unavailable, therefore, these values should be considered approximate estimates rather than measured reservoir temperature (Awais et al., 2019). Salinity related features were assessed using electrical conductivity, which ranged from 3.37–14.12 mS/cm (Table 2). Immediately after collection, all samples were stored on dry ice and then transported to the laboratory under refrigerated conditions for subsequent molecular analyses (Li et al., 2023).

**Table 1:** Sample group, sample ID, geological formation, and depth interval.

Sample group	Sample ID	Formation	Depth interval
Group 1	NW11-1 to NW11-10	Lockhart	3,780–3,925 m
Group 2	NW11-11 to NW11-15	Hangu	4,170–4,240 m
Group 3	NW11-16 to NW11-20	Lumshiwal	4,245–4,315 m

**Table 2:** Physicochemical characteristics of selected petroleum core samples from the Nashpa Oil Field.

Samples	pH	EC (mS/cm)	Phosphorus (PPM)	Nitrate Nitrogen (mg/kg)	Chromium (PPM)	Cadmium (PPM)	Lead (PPM)
Control	7.86	0.48	2.17	19.27	83.46	0.73	28.35
NW11-1	8.6	11.53	0.34	14.54	73.38	0.45	25.56
NW11-5	8.54	13	2.64	12.32	87.35	0.43	20.47
NW11-10	8.78	14.12	0.69	12.3	75.57	2.11	37.03
NW11-13	8.55	7.29	5.41	14.36	67.12	2.08	45.53
NW11-15	8.66	5.72	4.09	13.19	82.62	2.05	21.39
NW11-17	8.47	3.37	5.68	16.1	74.54	0.23	11.23
NW11-20	8.88	11.84	3.79	12.49	50.56	0.92	23.56

### Physicochemical analysis

Physicochemical characterization of selected reservoir-derived samples was performed to determine pH, electrical conductivity (EC), phosphorus, nitrate nitrogen, chromium, cadmium, and lead concentrations (Table 2). Prior to analysis, the samples were homogenized and prepared according to standard laboratory procedures. The pH and EC of samples were measured using calibrated pH and conductivity meters, whereas phosphorus and nitrate nitrogen concentrations were determined using standard spectrophotometric methods. Chromium, cadmium, and lead concentrations were determined following acid digestion of the samples using standard laboratory procedures for elemental analysis. Analytical procedures were carried out according to established standard methods for soil chemical analysis (Keeney and Nelson, 1982; Burt, 2004). The measured physicochemical parameters were used to provide environmental context for interpreting the thermotolerant, halotolerant, nitrate-reducing, and hydrocarbon-transforming genomic traits identified in the reservoir microbiome.

Nitrate-reducing bacteria was primary focus because nitrate injection is commonly used as a strategy to control reservoir souring and suppress sulfide production by sulfate-reducing bacteria. Therefore, understanding nitrate-reducing bacteria is important for evaluating biotechnological applications in petroleum reservoirs (Youssef et al., 2009).

### Genome-resolved metagenomic recovery

#### DNA extraction

Genomic DNA was extracted from petroleum reservoir core samples using the DNeasy PowerSoil Kit (Qiagen, Hilden, Germany) according to the manufacturer’s instructions. DNA concentration and purity were assessed using a NanoDrop ND-1000 spectrophotometer (NanoDrop Technologies, Wilmington, DE, USA). Purified DNA samples were stored at −20 °C until further processing (Pacwa-Płociniczak et al., 2020).

#### Library preparation, sequencing, and MAG recovery

Sequencing libraries were prepared using the Illumina TruSeq DNA Library Preparation Kit according to the manufacturer’s instructions. Shotgun metagenomic sequencing was performed by Microsynth on an Illumina NovaSeq platform using a paired-end 2 × 150 bp sequencing strategy (Frey et al., 2022).

#### Read quality control and assembly

Raw sequencing reads were assessed for quality using FastQC v0.11.9. Adapter sequences and low-quality reads were removed using standard quality-filtering procedures. High-quality reads were subsequently assembled *de novo* using MEGAHIT v1.2.9. Contigs shorter than 2,000 bp were excluded from further analyses prior to genome binning and downstream genome-resolved metagenomic analyses.

#### Genome binning, refinement and quality filtering

QUAST was used to assess the quality of the assembly in terms of N50, L50, total length, number of contigs, and GC content. MetaBAT2 and MaxBin2 were used to bin contigs into putative genome bins, which were subsequently refined using the DAS Tool. The completeness and contamination of the recovered MAGs were subsequently assessed using CheckM (Jensen et al., 2024; Mallawaarachchi et al., 2020; Rajeev et al., 2023). Additionally, GTDB-Tk 2.6.1 was used for taxonomic classification of MAGs.

#### Functional analysis

Nitrate-reduction genes (e.g., *narGHIJ, nirS/nirK,* and *norBC*) were identified from Prokka versions up to 1.14.6 + galaxy1 annotations and/or KO assignments from KEGG mapping, including map00190, map00362, map00623, map00626, map00630, and map00910. Functional annotations were further screened to identify genes associated with hydrocarbon transformation, respiratory pathways functioning under oxygen-limited conditions (including microaerophilic respiration and nitrate-linked respiration), aromatic compound degradation, and cellular stress-response mechanisms relevant to petroleum reservoir environments. Data summarization was visualized in RStudio via ggplot2 and dplyr (Bariya et al., 2025; Rout et al., 2022; Ruiz-Perez et al., 2021).

### Statistical analysis

Because the study was based on a limited number of core-derived samples and lacked independent biological replication for each depth/treatment category, the statistical results were interpreted cautiously. Community patterns are presented primarily as exploratory and descriptive. Data processing was performed by MEGAHIT, MetaBAT, CheckM, Prodigal/Prokka, GTDB-Tk 2.6.1, 1.14.6 + galaxy1 and KEGG. Data summarization and visualization were carried out in R Studio via ggplot2 and dplyr.

## Results

PCoA analysis (Supplementary Figure S1) showed differences in microbial community composition among samples. NW 11–17 and NW 11–20 clustered together as did NW 11–1 and NW 11–5, indicating similar community structures. NW 11–10 and NW 11–15 also grouped closely, whereas, NW 11–13 showed greater similarity to the control sample. These patterns suggest spatial variation in microbial community composition across the sampling sites (Little et al., 2024).

### Physicochemical characteristics and geochemical context of samples for MAGs recovery

The physicochemical characteristics of representative petroleum reservoir core samples are presented in Table 2. The analysed samples exhibited alkaline conditions, with pH values ranging from 8.47 (NW11-17) to 8.88 (NW11-20), compared with a pH of 7.86 in the control sample (natural surface soil from an uncontaminated area outside the oil filed used to compare the physicochemical characteristics and microbial properties of the petroleum reservoir associated samples.). Electrical conductivity (EC) varied substantially among samples, ranging from 3.37 to 14.12 mS/cm, indicating differences in salinity-related characteristics across the sampled cores. Phosphorus concentrations ranged from 0.34 to 5.68 ppm, whereas nitrate nitrogen concentrations varied between 12.30 to 16.10 mg/kg in reservoir samples.

Chromium was detected in all analysed samples, with concentrations ranging from 50.56 to 87.35 ppm. Cadmium concentrations varied between 0.23 and 2.11 ppm, while lead concentrations ranged from 11.23 to 45.53 ppm. Overall, the reservoir core samples displayed variable physicochemical and geochemical characteristics that provide an environmental context for the interpretation of the recovered metagenome-assembled genomes (MAGs) and their predicted functional potential.

### Genome-resolved functional potential (shotgun MAGs)

#### Metagenome binning and genome reconstruction statistics

A total of 402 MAGs were recovered. Of these, 216 MAGs met high-quality criteria defined by ≥90% completeness and ≤5% contamination and were retained for primary taxonomic and functional interpretation. Medium-quality MAGs were used only for supplementary overview, while low-quality MAGs were excluded from functional claims.

#### Taxonomic composition of high-quality MAGs

Based on GTDB classification, the 216 high-quality MAGs were distributed across multiple bacterial phyla (Table 3), with clear dominance of *Pseudomonadota* and *Actinobacteria. Pseudomonadota* were affiliated with approximately 60–65% of the recovered high-quality MAGs and included genera such as *Pseudomonas*, *Alcanivorax*, *Roseovarius*, *Marinobacter*, *Paracoccus*, and *Halomonas*. *Actinobacteriota* accounted for approximately 15–18% of the MAGs and were represented primarily by *Streptomyces* and *Nocardioides*. Additional phyla included *Bacteroidota, Planctomycetota,* and *Verrucomicrobiota,* whereas several candidate and uncultivated taxa were detected at lower relative abundances.

**Table 3:** Phylum-level distribution of recovered high-quality MAGs.

Phylum	Representation (%)	Representative genera
Pseudomonadota	~60–65%	*Pseudomonas, Alcanivorax, Roseovarius, Marinobacter, Paracoccus, Halomonas*
Actinobacteriota	~15–18%	*Streptomyces, Nocardioides*
Bacteroidota	~5–8%	*Wenzhouxiangella*
Planctomycetota	~4–6%	*Gimesia, Phycisphaera*
Verrucomicrobiota	~3–5%	*Mucisphaera*
Other minor phyla	~ < 5%	Candidate and Uncultivated taxa

Selected MEOR-relevant MAGs exhibited high completeness (>93%) and low contamination (<4%), indicating good genome quality for downstream taxonomic and functional analyses. Genome sizes ranged from 3.02 to 4.31 Mb, GC contents ranged from 59 to 71%, and contig N50 values varied among the recovered genomes (Table 4). Representative high-quality MAGs were affiliated with *Azospirillum thermophilum*, *Thermohalobaculum* sp., *Alcanivorax* sp., *Pseudomonas aeruginosa*, *Streptomyces* sp., and *Erwinia* sp.

**Table 4:** Quality metrics of representative high-quality MAGs carrying hydrocarbon transformation, nitrate reduction, low oxygen-repiratory metabolism, and stress response genes.

MAGs ID	GTDB Species	Completeness (%)	Contamination (%)	Contig N50	Genome Size (bp)	GC Content (%)
NW11-2_bins_40	*Azospirillum, thermophilum*	98	2.96	26,197	3,101,119	70
NW11-5_bins_54	*Thermohalobaculum* sp.	93.69	3.48	10,125	3,244,858	71
NW11-3_bins_32	*Alcanivorax* sp.	99.94	3.83	123,454	4,313,987	59
NW11-6_bins_52	*Pseudomonas aeruginosa*	99.99	1.24	87,742	4,305,471	60
NW11-4_bins_8	*Streptomyces* sp.	94.84	1.06	33,057	3,022,365	70
NW11-3_bins_20	*Erwinia* sp.	98.89	3.31	270,164	3,580,414	69

### Functional potential of high-quality MAGs

#### Hydrocarbon transformation genes

**Figure 1:**
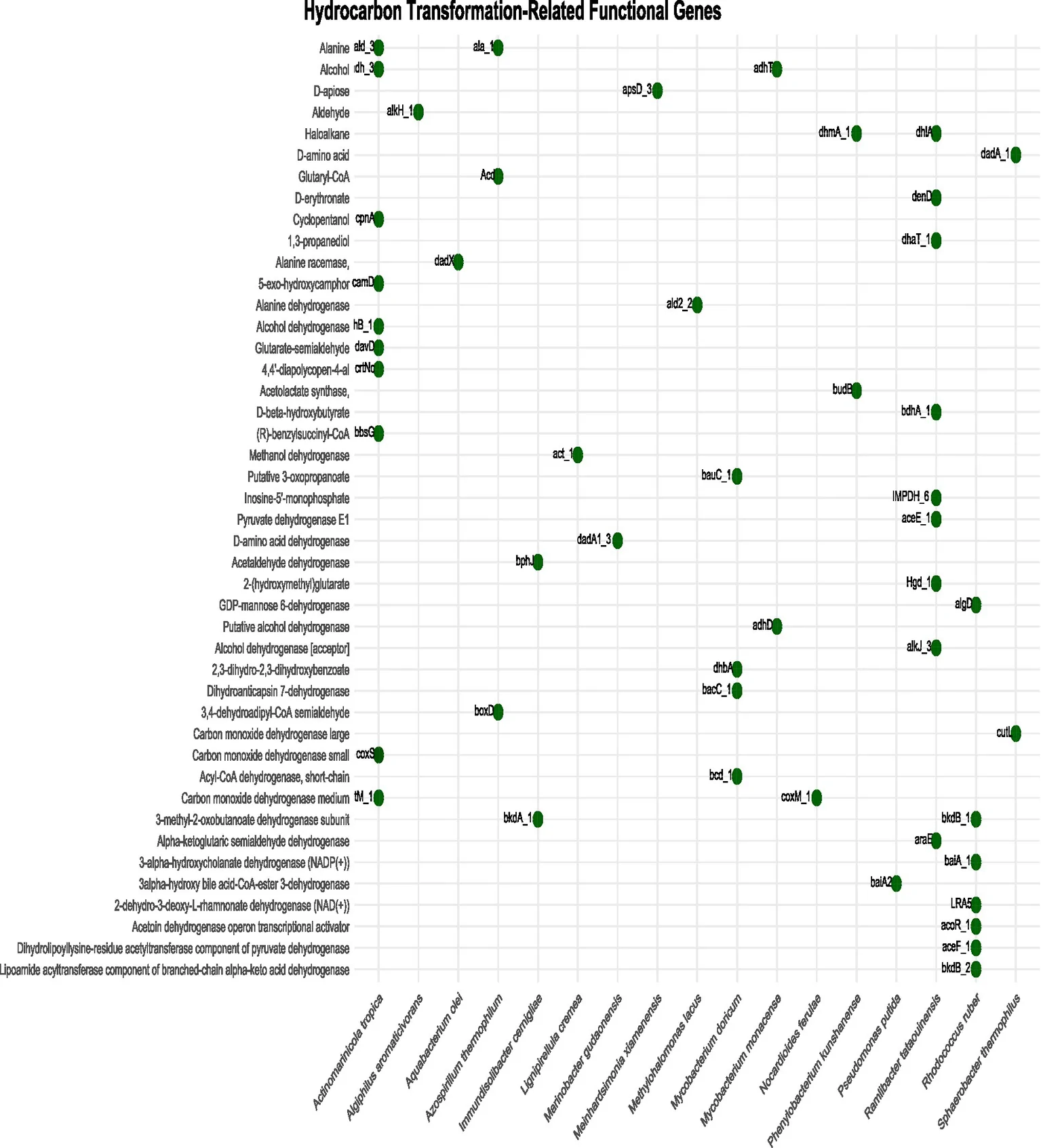
Distribution of functional genes associated with hydrocarbon/aromatic transformation, stress response, nutrient acquisition, and central metabolism. Only genes encoding monooxygenases, dioxygenases, and aromatic ring cleavage enzymes were considered direct indicators of hydrocarbon degradation potential. Genes encoding alcohol dehydrogenases, aldehyde dehydrogenases, acyl-CoA dehydrogenases and other general oxidoredcutases were treated as supportive metabolic genes because they may function in multiple pathways and are not specific to hydrocarbon degradation.

Predicted genes related to petroleum hydrocarbon transformation (Figure 1) were detected in a limited subset of high-quality MAGs rather than across the whole recovered community. Alkane monooxygenase related genes were uncommon, with *alkB* (alkane monooxygenase) and related alkane hydroxylase genes detected in 3 MAGs and contribute to the initial aerobic oxidation of alkane into alocohols and recognized as biomarker for aerobic alkane degradation, while additional genes potentially involved in alkane transformation included *alkB1, alkB2* (alkane monooxygenase isoforms), *ladA, alma* (long chain alkane monooxygenase), *alkH* (aldehyde dehydrogenase), *alkL* (alkane uptake protein), *alkJ* (alcohol dehydrogenase) oxidizes alcohol intermediates, *alkT* (rubredoxin reductase) supplying electrons to *AlkB*, and Cytochrome P450 monooxygenases (*CYP*-family genes) catalyze the oxidation of diverse hydrocarbon substrates and contribute to the aerobic transformation of petroleum hydrocarbons were each detected in 1 MAG (Guo et al., 2023; Wang and Shao, 2012; Feng et al., 2007; Van Beilen and Funhoff, 2007; Rojo, 2009; Wentzel et al., 2007). Alkane oxidation and associated downstream alcohol/aldehyde dehydrogenases (*adh* (alcohol dehydrogenase), *ald* (aldehyde dehydrogenase), *acd* (acyl-CoA dehydrogenase)) were detected in a limited number of genomes. *β*-Oxidation genes, e.g., *bcd* (butyryl CoA dehydrogenase) were co detected with initial monooxygenases in selected taxa. Aromatic degradation markers were also taxon specific, with the *bph* (biphenyl/aromatic degradation genes) detected in 5 MAGs and hca genes (phenylpropionate/hydroxycinnamate degradation) in 4 MAGs (Hirose et al., 2019; Fujihara et al., 2023), while *dmp, cat* (catechol ring cleavage pathway), *mhp* (hydroxyphenylpropionate degradation) and related dioxygenase genes were each detected in 2 MAGs. The *hca* and *mhp* gene clusters have been described in bacterial aromatic-compound catabolism, particularly phenylpropionate and hydroxyphenylpropionate degradation pathways, whereas *cat* genes are linked with catechol ring-cleavage steps in central aromatic metabolism (Xu and Zhou, 2020; de Sousa Júnior et al., 2025). Core alkane monooxygenase (*alkB, CYP153*) and aromatic ring-cleavage genes were detected in MAGs affiliated with *Rhodococcus, Pseudomonas, Marinobacter*, and *Nocardioides*. Therefore, these results indicate selected hydrocarbon-transformation potential rather than broad enrichment across the reservoir microbiome (Table 5).

**Table 5:** Distribution of functional genes associated with hydrocarbon transformation and anaerobic respiration among recovered high-quality MAGs.

Functional category	Gene Family	Number of MAGs carrying genes	Percentage of MAGs (%)
Alkane degradation	alkB	3	1.39
Alkane degradation	alkB1, alkB2	1	0.46
Alkane degradation	alkH, alkJ, alkT, cyp/CYP-family genes	1	0.46
Alkane degradation	alkB1_1, alkB2_1	2	0.93
Alcohol and aldehyde conversion	adh_3, adhB_1, alkH_1, exaA, and other alcohol/aldehyde dehydrogenases	11	5.09
β-oxidation	acyl-CoA dehydrogenase/Acd	1	0.46
Aromatic degradation	bph	5	2.31
Aromatic degradation	hca	4	1.85
Aromatic degradation	cat genes	2	0.93
Aromatic degradation	mhp genes	2	0.93
Aromatic ring cleavage	bph genes, e.g., bphO; catD_1; hca genes, e.g., hcaB_10	10	4.63
Anaerobic respiration	nar operon	7	3.24
Anaerobic respiration	nir genes	3	1.39
Anaerobic respiration	nor genes	2	0.93
Anaerobic respiration	fdh family	2	0.93

#### Nitrate-reduction genes

**Figure 2:**
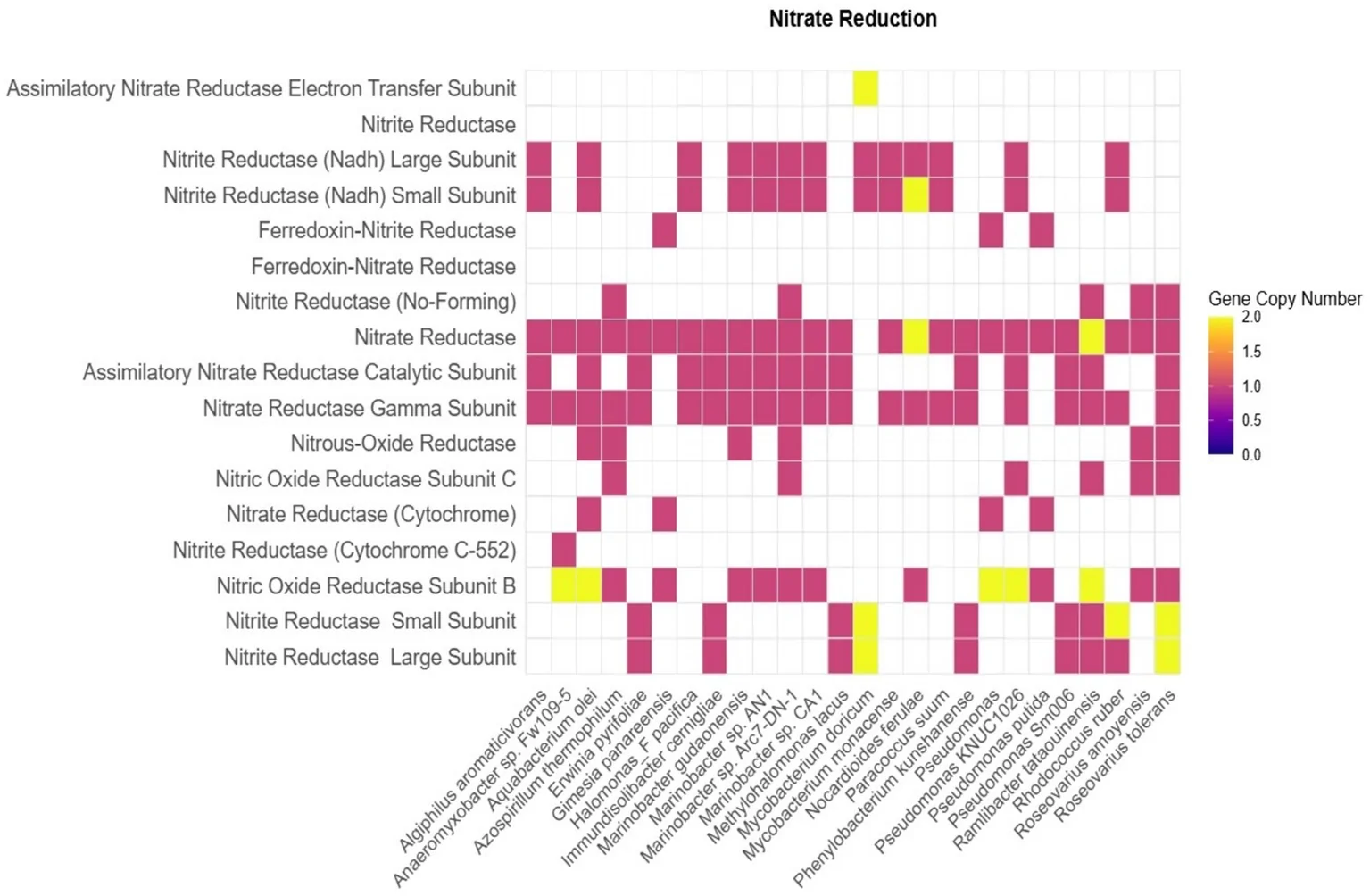
Heatmap showing the normalized relative distribution of functional genes across the top 25 microbial species identified in the assembled metagenomes. Microbial species are shown on the vertical axis, and functional gene categories are shown on the horizontal axis. Color intensity indicates normalized gene distribution.

A heatmap (Figure 2) shows the distribution of nitrate- and nitrite-reduction genes among the recovered high-quality MAGs. Clear variation was observed among taxa, indicating functional specialization within the nitrate-reduction pathway. *Roseo*var*ius*, *Pseudomonas*, and certain *Marinobacter* MAGs exhibited a greater diversity of nitrate-reduction genes than other taxa. Among the selected MEOR-relevant high-quality MAGs, nitrate-reducing genes of the *nar* operon were detected in seven high-quality MAGs affiliated with *Pseudomonas*, *Marinobacter*, and *Roseovarius*. Among these, the operon was most complete and structurally conserved in the *Pseudomonas*-affiliated genome (NW2-1-21), whereas partial or variant configurations were observed in the other MAGs.

The *narGHIJ* operon genes (nitrate reductase system) encodes the membrane bound respiratory nitrate reductase system in which *NarG* and *NarH* form the catalytic nitrate reductase complex involved in nitrate reduction to nitrite, whereas *NarI* anchores the complex to the membrane and *NarJ* helps in enzyme maturation were the most frequently detected anaerobic respiration genes (3.24% of MAGs), whereas *nir* genes encoded nitrite reductases particularly *nirS/nirK* type enzymes converts nitrite to nitric oxide during denitrification were detected in three MAGs, and *nor* genes and *fdh-*family genes may support anaerobic respiration by oxidizing formate and supplying electrons to respiratory pathways were each detected in two MAGs (Pold et al., 2024; Intrator and Ward, 2025; Durand and Guillier, 2021). Genes encoding respiratory nitrate reductase, nitrite reductase, nitric oxide reductase, formate dehydrogenase, cytochrome bd oxidase, and benzoyl-CoA reductase-related functions were detected in selected high-quality MAGs. Benzoyl CoA reductase genes are not nitrate reduction genes but are linked to anaerobic respiration of aromatic compound through the benzoyl CoA pathway (de Sousa Júnior et al., 2025; Borisov et al., 2021) These findings indicate that multiple taxa may contribute to nitrate-reduction potential within the reservoir environment, with *Pseudomonas* carrying the most complete genes associated with nitrate-reduction pathway.

**Figure 3:**
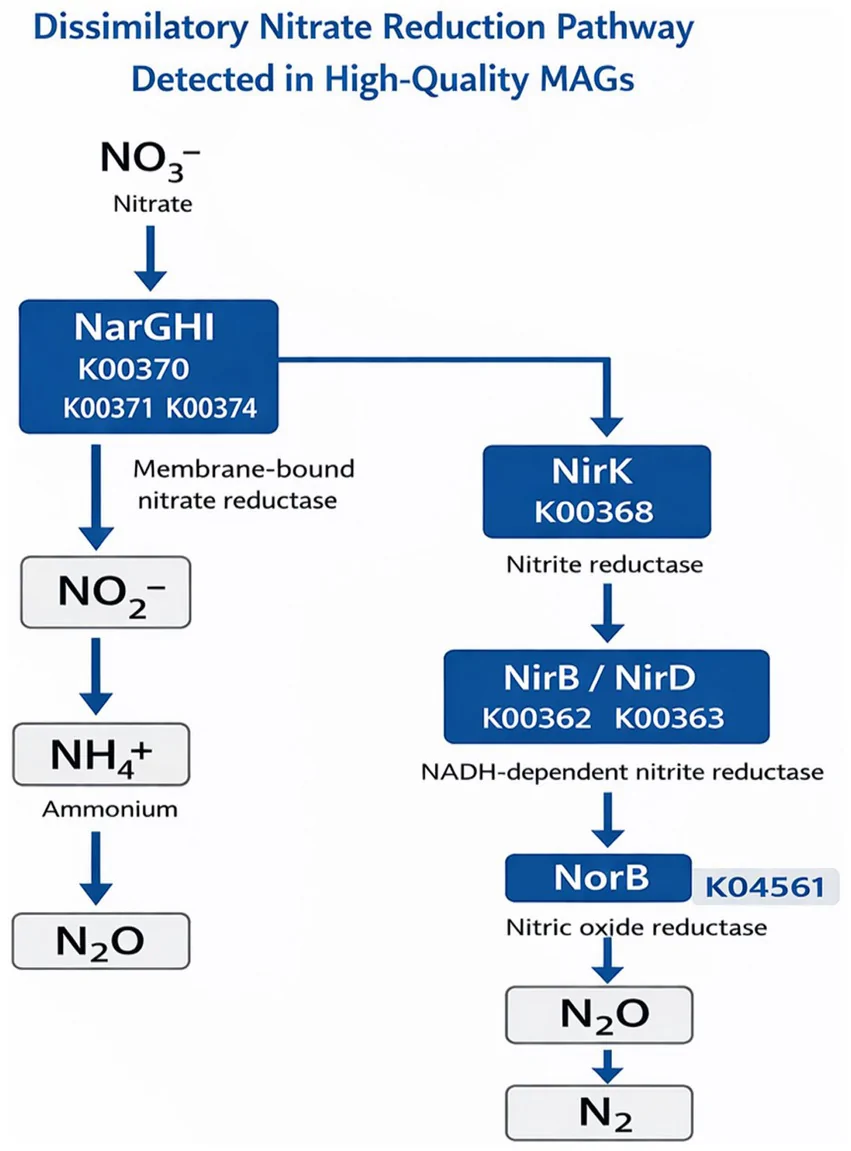
Assimilatory and dissimilatory nitrate reduction pathways (KEGG map00910).

KEGG map00910 analysis (Figure 3) further revealed that nitrate-reduction genes were unevenly distributed among the recovered MAGs. In addition to *Pseudomonas* and *Marinobacter*, MAGs affiliated with *Halomonas* and *Idiomarina* also carried multiple nitrate-reduction markers, including nitrate reductase, nitrite reductase, and nitric oxide reductase subunits. Certain genes, such as genes coding nitric oxide reductase subunit B and cytochrome C-552 nitrite reductase, were restricted to specific taxa, whereas nitrate reductase and assimilatory nitrate-reduction electron-transfer components were more broadly distributed. These patterns suggest functional partitioning of nitrate-transformation processes among reservoir-associated microorganisms.

Genes associated with nitrate-linked respiration, microaerophilic metabolism, and anaerobic aromatic-compound transformation were distributed unevenly among the recovered MAGs. Genes associated with anaerobic aromatic compound metabolism were represented primarily by benzoyl CoA reductase which is a key step in the anaerobic degradation of aromatic compounds through reduction of the benzoyl CoA intermediate. However, no benzyl succinate synthase genes (responsible for anaerobic activation of toluene and related aromatic hydrocarbons) were detected in recovered MAGs. Because the analysis is based on gene presence and pathway annotation, these findings should be interpreted as predicted metabolic potential rather than evidence of active oxygen-limited metabolism *in situ*.

#### General heat stress-response and respiratory genes under oxygen limited conditions

**Figure 4:**
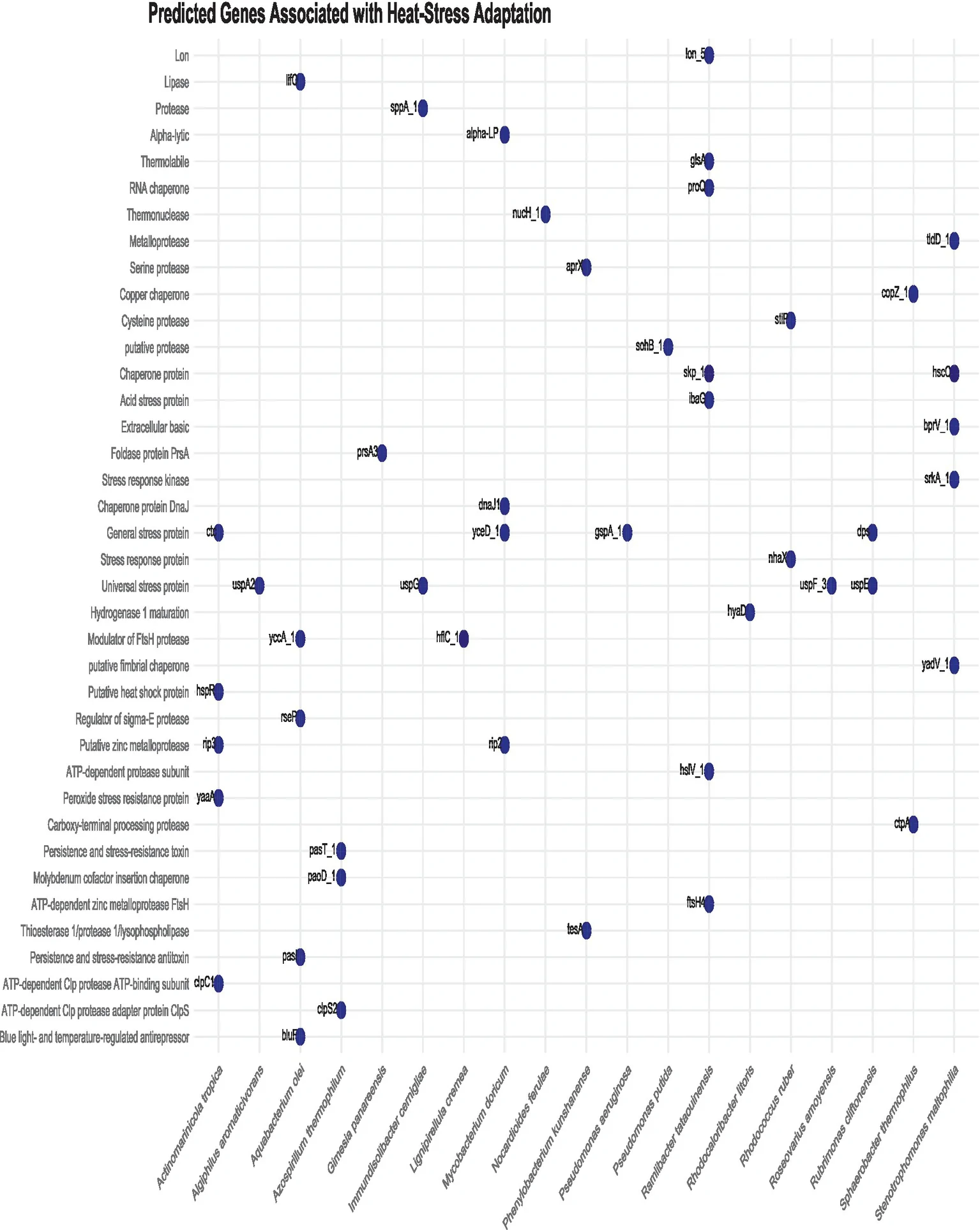
Distribution of genes related to stress response and adaptation. Distribution of genes involved in molecular chaperon systems, protein quality control, heat shock response, oxidative stress protection and general stress adaptation across selected MAGs.

Genes associated with the heat stress response (Figure 4), including *dnaJ, dnaK encoding the Hsp 70 molecula chepron and its co chaperon dnaJ which refolds misfolded proteins generated during heat oxidative and other general stress, groEL enodes the groEL chaperonin that promotes correct protein folding and prevents aggregation of damages proteins, ATP dependent protease genes including clp, lon, hslV, ftsH* and universal stress proteins encode components of bacterial protein quality control and degrading damaged proteins, were detected in selected MAGs. Genes related to stress adaptation, including *uspA, uspB, uspF encode universal stress protein involved in adaptation nutrient limitation, yajL encodes a DJ family protein involved in protecting proteins from oxidative damage, dps encodes a DNA binding ferritin like protein that protects DNA from oxidative damage,* and *katE encodes catalase involved in hydrogen peroxide detoxification,* were also detected in selected MAGs. Several heat shock proteins and general stress response genes including metalloproteases, amino acid dehydrogenases, blue-light/temperature antirepressors, and ATP-dependent proteases, were identified (Figaj, 2025; Park and Seo, 2015). Notably, MAGs affiliated with *Pseudomonas, Rhodococcus, Mycolicibacterium,* and *Nocardioides* contained a greater diversity of stress-response genes. Similar gene complements were observed in MAGs affiliated with *Sphaerobacter thermophilus, Rhodococcus ruber,* and *Ramlibacter tataouinensis*.

**Figure 5:**
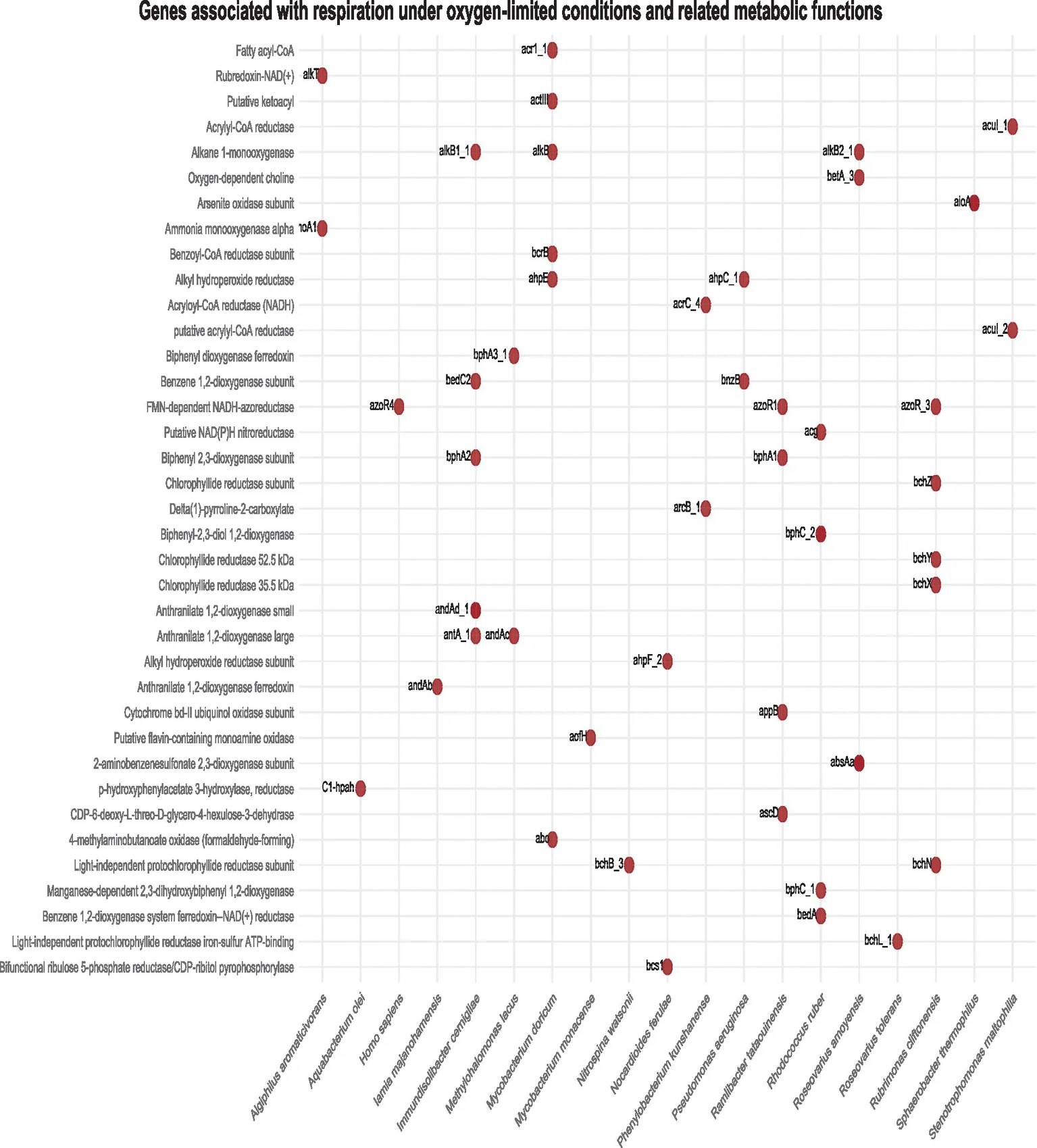
Distribution of functional genes associated with respiration under oxygen limited conditions and microaerophilic metabolism across microbial taxa identified in the metagenomic dataset. Points represent detected genetaxon associations related to oxidative stress response, reductive metabolism, and electron transport.

In this study, oxygen limited respiratory metabolism refer to predicted respiratoy pathways that enable microorganisms to conserve energy when oxygen availability is limited, through high affinity cytochrome bd oxidase (*cydAB*) and nitrate linked respiratory genes (*narGHIJ, nirS, and norBC*). Other genes shown in Figure 5 are presented because they co-occurred within the KEGG functional annotation of the analyzed MAGs and therefore provide broader metabolic context rather than representing low-oxygen respiration directly. Genes associated with respiration under oxygen limited conditions (Figure 5) were detected in selected high-quality MAGs rather than across the whole recovered community. Genes associated with respiration under oxygen limited conditions, redox balance, and aromatic-compound transformation were distributed unevenly among taxa (Figure 5). The cytochrome *bd* oxidase marker *cydB_3/cydAB in oxidative phosphorylation pathway* (K02293) and NADH dehydrogenase-associated genes (Supplementary Figure S2) (*encode the cytochrome bd terminal oxidase that supports aerobic respiration under low oxygen respiration*) was detected in one MAG, representing 0.46% of high-quality MAGs. Identified genes are associated with respiratory pathways functioning under reduced oxygen availability rather than implying a specific oxygen concentration, which was not measured in this study (Wang et al., 2021; Hossain et al., 2024; Torres-Beltrán et al., 2025). Nitrate-linked respiration genes were also limited in distribution, with *narGIHJ (nitrate reductase system)* detected in three MAGs, *nirS* in one MAG, and *norBC (encode the nitric oxide reductase which catalyzes the reduction of nitric oxide to nitrous oxide during deniftrification)* in two MAGs (Pold et al., 2024). A benzoyl-CoA reductase marker, *bcrB (key enzyme of anaerobic aromatic hydrocarbon degradation)*, was detected in one MAG, representing 0.46% of high-quality MAGs (Godínez-Pérez et al., 2024) (Table 6). These results indicate that only a limited subset of MAGs encoded markers associated with low oxygen- respiration and anaerobic aromatic-compound transformation. Because this analysis is based on gene presence and KEGG annotation, these findings should be interpreted as predicted metabolic potential rather than evidence of active oxygen-limited metabolism or active anaerobic hydrocarbon degradation (Frey et al., 2022; Hidalgo et al., 2021; DeCastro et al., 2016). KEGG pathway reconstruction further supported these findings by identifying genes associated with oxidative phosphorylation (map00190), aromatic-compound degradation (map00362, map00623, map00626), and glyoxylate metabolism (map00630) (Supplementary Figures S2, S3a,b, S4) in the recovered high-quality MAGs. These pathways included genes involved in aromatic compound transformation (Supplementary Figures S3a,b) included *dmpH, adhP, yiaY, frmA, adhE, hcaCDEF* (Okoye et al., 2024; Bayatian et al., 2025; Shebl et al., 2024; Mohapatra and Phale, 2021; Tian et al., 2025) and carbon/redox metabolism (Supplementary Figure S4, map00630) genes including *glcDE, gcvPHTL, fdhA, fdoG indicating predicted metabolic capabilities among the recovered MAGs* (Li et al., 2023; Abdul Halim et al., 2024; Tezuka and Ohnishi, 2014). As KEGG reconstruction was based on gene presence these pathways are interpreted as predicted metabolic potential rather than evidence of active *in situ* metabolic activity.

**Table 6:** Distribution of genes associated with low oxygenrespiratory metabolism in recovered high-quality MAGs.

Metabolic type	Genes	KEGG map	Number of MAGs carrying genes	Percentage of MAGs (%)	MAGs IDs	Conservative interpretation
Microaerophilic	cydAB marker	map00190	1	0.46	NW11-3_bins_11	Predicted Oxygen limited respiratory potential
Facultative anaerobic	narGIHJ	map00910	3	1.39	NW11-1_bins_59, NW11-1_bins_57, NW11- 1_bins_56,	Predicted nitrate reduction potential
Facultative anaerobic	nirS	map00910	1	0.46	NW11-1_bins_56	Predicted nitrite reduction potential
Facultative anaerobic	norBC	map00910	2	0.93	NW11-1_bins_39, NW11-1_bins_56	Predicted nitric oxide reduction potential
Strict anaerobic	benzoyl-CoA reductase	map00362	1	0.46	NW11-1_bins_20	Predicted anaerobic aromatic compound transformation potential

#### MEOR-relevant MAGs

Among the 216 high-quality MAGs, several taxa showed genomic features relevant to MEOR, including stress response, oxygen-limited metabolism, and hydrocarbon transformation potential. MEOR-relevant candidate MAGs were selected using combined, genome-quality and functional-evidence criteria rather than taxonomy alone. Only high-quality MAGs meeting the thresholds of ≥90% completeness and ≤5% contamination were considered for candidate selection, and they were required to encode genes associated with at least two functional trait groups relevant to microbial processes involved in MEOR, including hydrocarbon or aromatic-compound transformation, nitrate-linked metabolism, oxygen-limited or redox metabolism, and cellular stress response. Based on these criteria, selected MAGs affiliated with *Thermohalobaculum* spp., *Azospirillum thermophilum*, *Pseudomonas aeruginosa*, *Alcanivorax* spp., *Streptomyces* sp., *Erwinia* sp., and *Spiribacter* were retained as MEOR-relevant genomic candidates. The functional basis for candidate selection (Supplementary Table S1) shows that the *Alcanivorax*-affiliated MAG NW11-3_bins_32 encoded alkane and aromatic-transformation markers, including *alkB1, alkB2, alkJ, tmoA, xylG, hcaB,* and *bphF*, associated with nitrate reduction pathway and stress-response systems. Similarly, *Pseudomonas aeruginosa* NW11-6_bins_52 encoded nitrate-reduction genes, including *narG, narH, narI, narT,* and *napA,* as well as aromatic transformation markers, such as *ndoR, hcaB,* and *pnpB*. Other candidate MAGs contained combinations of nitrate-reduction, oxygen-limited respiratory, aromatic-transformation, and stress-response genes. These MAGs are therefore interpreted as genomic candidates possessing predicted traits for hydrocarbon transformation, nitrate metabolism, respiration under oxygen limited conditions metabolism and cellular stress adaptation which may support microbial process relevant to MEOR under petroleum reservoir conditions only and not as experimentally confirmed MEOR-performing organisms.

## Discussion

This study provides one of the first genome-resolved functional insights into microbial communities inhabiting deep subsurface petroleum cores from Pakistan using Shotgun metagenomic sequencing.

The observed separation and clustering of samples in the PCoA analysis indicate the heterogeneity in microbial community composition across the reservoir. Differences in local physicochemical conditions and nutrient availability may contribute to the distinct community structures observed among sampling locations. Similar patterns have been reported in petroleum associated environments, where environment influence microbial diversity (Gittins et al., 2023).

### Functional metabolic potential of microbial communities

#### Hydrocarbon transformation potential is taxon-specific

Hydrocarbon-degradation markers were detected only in selected high-quality MAGs, suggesting that hydrocarbon transformation was concentrated within specialized thermotolerant bacteria rather than being a community-wide trait. The presence of *alkB, bph* and dehydrogenase families indicates genomic potential for alkane oxidation, aliphatic and aromatic hydrocarbon transformation; however, these markers should be interpreted as evidence of candidate hydrocarbon-associated genomes, not direct proof of active *in situ* degradation. According to (de Sousa Júnior et al., 2025) alkane monooxygenases and aromatic ring cleavage enzymes represent key functional markers involved in petroleum hydrocarbon biodegradation pathways. However, several dehydrogenase genes detected in the present study may participate in multiple metabolic pathways and therefore should be interpreted as supporting metabolic functions rather than exclusive hydrocarbon degradation markers. This pattern agrees with Vázquez Rosas Landa et al. (2023), who used DNA-SIP metagenomics with ^13^C-n-hexadecane and showed that several SIP-enriched MAGs encoded alkane-oxidation genes such as *AlkB, AlkT* and *AlkG,* including previously overlooked hydrocarbon-degrading taxa such as *Lentibacter* and *Dokdonia*. Similarly, Xu et al. (2022) reported a shale oil-associated *Stenotrophomonas maltophilia* strain with long-chain alkane-degrading ability, biosurfactant production and alkane-related genes, supporting the view that selected bacteria can combine hydrocarbon transformation with adaptive traits that improve hydrocarbon bioavailability and survival under stress. Therefore, the MAGs identified in the present study highlight distinctive functional adaptations that may support hydrocarbon transformation, stress tolerance, and hydrocarbon utilization under petroleum reservoir conditions.

#### Putative heat stress adaptation and thermotolerance potential

Functional annotation of the MAGs revealed stress adaptation and protein quality control genes, including *hsp20*, *hsp70*, *hslV*, *clp* proteases, *uspA/F*, acid stress proteins, metalloproteases, *dnaK* (encoding an Hsp70 molecular chaperone together with its cognate *dnaJ*), protects protein damage during environmental stress, *groEL* (encoding a chaperonin that prevents protein aggregation during denaturation), and *prsA* (encodes a membrane-associated foldase that promotes the stability of secreted proteins under heat stress) (Figaj, 2025; Park and Seo, 2015). These genes may support bacterial survival under harsh petroleum-associated conditions by maintaining protein stability, repairing misfolded proteins and reducing stress-related cellular damage. The detected stress-response genes are commonly associated with protein folding, protein quality control, and cellular stress-response functions. Universal stress-response genes such as *uspA, uspB, uspF (encode universal stress protein involved in adaptation nutrient limitation),* and *yajL (encodes a DJ family protein involved in protecting proteins from oxidative damage)* have been linked with survival under adverse environmental conditions (Luo et al., 2023; El Qaidi et al., 2021), whereas peroxide-resistance genes such as *dps* (*encodes a DNA binding ferritin like protein that protects DNA from oxidative damage*)and *katE* (*encodes catalase involved in hydrogen peroxide detoxification*) contribute to protection against oxidative stress (Yuan et al., 2021; Williams and Chatterji, 2023). The observed distribution of chaperone systems, proteolytic systems, oxidative-stress defenses, and related stress-response genes suggests potential mechanisms that may support microbial survival under petroleum reservoir conditions. However, based on regional geothermal gradients, approximate reservoir temperatures for the sample intervals were taken, rather than measured reservoir temperature, these genes should be interpreted as indicators of putative stress-adaptation potential rather than direct evidence of thermotolerance (Roncarati and Scarlato, 2017; Fourie and Wilson, 2020; Alam et al., 2021; Jeong et al., 2020; Illigmann et al., 2021; Aljghami et al., 2022; Kirthika et al., 2023; Schroeder and Jonas, 2021). Similar stress response features have been reported by Saxena et al. (2016), who used 16S rRNA amplicon and shotgun metagenomic sequencing of microorganisms from Indian hot springs ranging from 43.5–98 °C and reported hydrocarbon-degradation pathways, including benzoate, xylene, toluene and benzene degradation, along with survival-related metabolic features of thermophilic communities dominated by taxa such as *Pseudomonas stutzeri* and *Acidovorax.* Therefore, the stress-response genes detected in the present MAGs may represent adaptive genomic traits that help selected thermotolerant bacteria maintain protein stability and hydrocarbon-transforming potential under petroleum-associated thermal stress.

#### Metabolic potential for respiration under oxygen-limited conditions and nitrate-reduction metabolism

Genes associated with nitrate-linked, microaerophilic and oxygen-limited metabolism were detected in selected high-quality MAGs, with *narGHIJ* being more frequent, while *nirS/nirK, norBC* and *fdhA* occurred in fewer genomes, suggesting taxon-specific metabolic flexibility rather than a community-wide anaerobic phenotype. The co-occurrence of *nar/nir/nor/fdh, cydAB* and oxygen-independent reductases, including benzoyl-CoA reductase, indicates that some Nashpa reservoir taxa may possess genomic features for nitrate-linked respiration, low-oxygen survival and anaerobic aromatic-compound transformation; however, these annotations should be interpreted as metabolic potential, not direct evidence of active *in situ* anaerobic hydrocarbon degradation. The detection of benzoyl CoA suggests the presence of microorganism capable of processing aromatic intermediates through the reduction of benzoyl CoA pathway under low oxygen conditions. However, the absence of benzylsuccinate synthase genes indicates that direct anaerobic activation of aromatic hydrocarbons could not be confirmed. Similar observations have been reported in petroleum associated microbial communities where downstream aromatic degradation pathways are present despite the absence of canonical hydrocarbon activation genes (Godínez-Pérez et al., 2024; Dong et al., 2019). Assimilatory and dissimilatory nitrate reduction are two fundamentally different microbial pathways with distinct roles in the nitrogen cycle and implications for MEOR. Assimilatory nitrate reduction converts nitrate (NO₃^−^) to ammonium (NH₄^+^) for incorporation into biomass and cellular nitrogen-containing molecules, supporting growth without significant energy conservation for respiration. In contrast, dissimilatory pathways serve mainly for energy generation under anaerobic conditions: denitrification sequentially reduces nitrate to gaseous N_₂_ or N_₂_O, resulting in loss of fixed nitrogen from ecosystems, while dissimilatory nitrate reduction to ammonium (DNRA) reduces nitrate to ammonium, thereby retaining bioavailable nitrogen in the environment rather than releasing it as gas. This distinction affects the ecosystem nitrogen balance, with DNRA promoting nitrogen retention and maintaining nitrogen availability for microbial communities, whereas denitrification contributes to nitrogen loss. In MEOR, stimulating dissimilatory nitrate reduction pathways, including DNRA and denitrification, provides alternative electron acceptors for anaerobic microbial metabolism under reservoir conditions. These pathways can also reduce reservoir souring by competing with sulfate-reducing microorganisms for substrates and generating inhibitory intermediates such as nitrite, thereby enhancing reservoir performance (Wang et al., 2024). This interpretation is consistent with Chen et al. (2024), who used 16S rRNA sequencing, metagenomics and hydrocarbon analysis in high-Arctic beach mesocosms and reported selected MAGs carrying hydrocarbon-degradation genes together with denitrification genes such as *narG, nirK, norB* and *nosZ,* highlighting metabolic versatility under oxygen-limited conditions. Similar functional specialization was reported by Goma-Tchimbakala et al. (2023) in gasoline/diesel-contaminated soils, where shotgun metagenomics identified dominant *Gordonia* and *Pseudomonas* populations with alkane and aromatic hydrocarbon pathways, and by Peng et al. (2024) who showed that a thermophilic petroleum-degrading consortium enriched from 55 to 70 °C compost could function in petroleum-contaminated soil at 55 °C. Our genomic analyses revealed that the Nashpa MAGs contain selected redox-adaptive and hydrocarbon-associated traits. These findings are consistent with previous reports describing the importance of such traits for microbial persistence and metabolic activity in oxygen-limited, hydrocarbon-rich reservoir environments (Goma-Tchimbakala et al., 2023; Peng et al., 2024).

#### Aromatic compound transformation and KEGG pathway reconstruction

KEGG pathway reconstruction identified genes and modules associated with aromatic hydrocarbon transformation, including benzoate, xylene, naphthalene and related pathways, with genes such as *dmpH, pcaC, pcaL, hcaE, hcaF, hcaC and hcaD* together with KEGG orthologues K13953/K00121, K01617 and K05708-K05710, suggesting that selected MAGs may transform naphthalene/methylnaphthalene-, catechol-, protocatechuate- and hydroxycinnamate-derived intermediates into central metabolites such as pyruvate, acetyl-CoA, fumarate and succinate. However, these annotations should be interpreted as genomic potential for aromatic hydrocarbon transformation, rather than direct evidence of complete *in situ* mineralization of BTEX or PAHs. This interpretation is supported by Sierra-García et al. (2014), who reported novel and fragmented aromatic hydrocarbon-degradation pathways in Brazilian petroleum reservoir metagenomes, and by Eze (2021), who showed that a diesel-enriched bacterial consortium contained 105 putative coding sequences involved in BTEX activation and catechol/alkyl catechol ring cleavage metabolism. Similar hydrocarbon-associated functional potential was reported by in gasoline- and diesel-contaminated soils, where shotgun metagenomics detected alkane and aromatic hydrocarbon pathways dominated by Goma-Tchimbakala et al. (2023) taxa such as *Gordonia* and *Pseudomonas*. In the present study, the detection of the glyoxylate pathway may further support carbon conservation and redox balance during assimilation of acetyl-CoA-derived intermediates, consistent with Chen et al. (2024) who described the glyoxylate cycle as an ancillary pathway of the TCA cycle that supports growth on acetyl-CoA-generating substrates rather than an independent anaerobic energy-generating route. Our metagenomic analysis provides insights into the potential metabolic capabilities of the microbial communities. It is important to note that all functional predictions are based on genome sequences and bioinformatic inference. These predictions are based on genomic annotation and have not been experimentally validated; actual metabolic activity under environmental conditions may differ. Future experimental studies are required to validate these predicted metabolic pathways and their relevance to biological systems.

### Limitations

Biological replication was not feasible due to the logistical and operational constraints associated with obtaining independent petroleum core samples from depths 3,770–4,315 m. Consequently, the results represent the analyzed samples and may not fully capture the spatial variability of the reservoir. The MEOR relevance of this dataset lies in the identification of candidate genomic traits, including hydrocarbon-transformation genes, nitrate-reduction genes, oxygen-limited metabolism markers, and stress-response systems. However, the study does not demonstrate biosurfactant production, hydrocarbon degradation, nitrate-reduction activity, or improved oil recovery. Therefore, the MEOR implications presented here should be considered hypotheses generated from genome-resolved metagenomic analyses and require future experimental validation through cultivation-based studies, functional assays, and reservoir-simulation experiments.

## Data Availability

The metagenomic sequence data generated and analysed during this study have been deposited in the NCBI Sequence Read Archive (SRA) under BioProject accession number PRJNA1094864 (SRP499294). The corresponding SRA accession numbers are available through the NCBI database.
