# Supplementary material for: Genome-resolved insights into hydrocarbon-transforming and nitrate-reducing microbial communities from deep petroleum reservoir cores of the Nashpa Oil Field, Pakistan
Source: Front Microbiol. 2026 Aug 11;17:1899441. doi: 10.3389/fmicb.2026.1899441 (PMC13503583; doi:10.3389/fmicb.2026.1899441)
Supplement: Supplementary file 1 [file Supplementary_File_1.docx]

**Genome-Resolved Insights into Hydrocarbon-Transforming and Nitrate-Reducing Microbial Communities from Deep Petroleum Reservoir Cores of the Nashpa Oil Field, Pakistan**

Sajida Tayyaba^1^**,** Arshia Amin^1*^, Sobia Zulfiqar^2^, Iftikhar Ahmed^2*^

^1^Department of Bioinformatics and Biosciences, Faculty of Health and Life Sciences, Capital University of Science and Technology Islamabad

^2^ National Culture Collection of Pakistan (NCCP), Land Resources Research Institute (LRRI), National Agricultural Research Centre, Islamabad, Pakistan

**Sobia Zulfiqar**

Email: sobia.cui@gmail.com

ORCID: 0009-0001-2658-0344

*** Corresponding author(s):**

**Arshia Amin**

Email: arshia.butt@cust.edu.pk

ORCID: 0000-0001-9074-4668

**Iftikhar Ahmed**

E-mail: iftikhar.ahmed@parc.gov.pk

ORCID: 0000-0003-0969-7927

Supplementary Table and figures

**Table (S1). Functional evidence supporting selection of MEOR-relevant candidate MAGs.**

| **Candidate MAG/Taxon** | **MAG IDs** | **GTDB species** | **Completeness (%)** | **Contamination (%)** | **Hydrocarbon/aromatic transformation genes** | **Nitrate reduction/ denitrification genes** | **Oxygen limited/redox genes** | **Stress response genes** |
| --- | --- | --- | --- | --- | --- | --- | --- | --- |
| *Thermohalobaculum spp.* | NW11-5_bins_54 | *Thermohalobaculum* spp. | 93.69 | 3.48 | alkB, catE, pht3/pht4, ophA1, hcaB, bbsG, dhmA | nirS, norB/norC, nosZ, nirQ | ccoP; nir/nor denitrification markers | dnaK, dnaJ, clpA/clpB/clpP/clpS, hslU/hslV, ftsH, hspQ, katG, uspF/uspG, lon |
| *Azospirillum thermophilum* | NW11-2_bins_40 | *Azospirillum thermophilum* | 98 | 2.96 | bphA3/bphF, hcaB/hcaC/hcaF, mhpB, nagG/nagI, pht3/pht4, ophA1, bbsG, bcd/fadH | napA, norV, nrtB/nrtC | ccoN1, norV | dnaK, dnaJ, clpA/clpB/clpP/clpS/clpX, hslU/hslV/hslR, ftsH, hspQ, lon |
| *Pseudomonas aeruginosa* | NW11-6_bins_52 | *Pseudomonas aeruginosa* | 99.94 | 3.83 | alkB1, alkJ, hcaE, hcaC, hcaB, catD_1, pnpA, bnzB, bbsG, ssuD, linB, dhaA/dhmA, fadE/acdA | napA, narB, nasD, nirD, norR, nosZ, nrtP | ccoN1/ccoN2/ccoP2, ccoO2, petA, nuo genes, fdoG/fdhD, ccm genes, NorR | dnaK, dnaJ, clpA/clpB/clpP/clpS/clpX, hslU/hslV/hslR, ftsH, htpG, hspQ, ibpA/ibpB, katG, ahpC/ahpF, uspA/uspE, lon |
| *Alcanivorax spp.* | NW11-3_bins_32 | *Alcanivorax* spp. | 99.99 | 1.24 | alkB1, alkB2, alkJ, tmoA, xylG, tphA1I, bphF, hcaB, mhpF, fadE/fadH, bcd, dhmA/dhaA | napA, nasA/nasD, nirD, norV, nrtD/nrtP | ccoN1/ccoP/ccoP2, appB | dnaK, dnaJ, clpA/clpB/clpP/clpS/clpX, hslU/hslV, ftsH, htpG, katG, ahpC/ahpF, uspA/uspF, lon |
| *Streptomyces sp.* | NW11-4_bins_8 | *Streptomyces* sp. | 94.84 | 1.06 | nagAb; dioxygenase-related markers including hmgA/hpd/kynA/kmo/amnC | napA, norR/norW, nosZ | napA/norR/norW nitrate/nitric oxide-related markers | dnaK, dnaJ, clpB/clpP/clpX, ftsH, lon, ahpE |
| *Erwinia sp.* | NW11-3_bins_20 | *Erwinia* sp. | 98.89 | 3.31 | alkB/alkB1, alkH, alkJ, hcaB, bbsG, bcd, fadE/fadH, linB, dhmA | narG/narH/narJ/narV/narT, nasA/nasC/nasD/nasE, nrtP, norR | cydB, ccoN1/ccoP2, fnr | dnaK, dnaJ, clpA/clpB/clpP/clpS/clpX, hslU/hslV/hslR, ftsH, htpG, katG, ahpC, uspF, lon |
| *Spiribacter* | NW11-2_bins_51 | *Spiribacter* | 99.26 | 1.64 | hcaB, pnpB, alkJ, dhaA/dhmA1, fadE/fadN | napA, nirS, nirQ, norB/norC, nrtD | cydA, ccoN1/ccoP2, fdhB1/fdhF/fdnH, fnr | dnaK, dnaJ, clpA/clpB/clpP/clpS/clpX, hslU/hslV/hslR, ftsH, htpG, hspQ, katG, ahpC, uspA/uspF/uspG, lon |

A

B

**Figure S1**. Principal coordinates analysis (PCoA) of microbial community composition based on (a) weighted UniFrac and (b) unweighted UniFrac distance matrices. Axis 1 and Axis 2 explain 18.5% and 15.0% of the variation in the weighted UniFrac analysis and 81.3% and 14.3% of the variation in the unweighted UniFrac analysis, respectively. Samples that cluster closer together share more similar phylogenetic community structures, whereas greater separation indicates increased differences in microbial community composition.

**Figure S2.** An illustration of the biological oxidative phosphorylation pathway and respiratory electron transport components linked to oxidative phosphorylation (KEGG map00190)

**3a**

**3b**

**Figure S3 (a, b).** Schematic representation of microbial petroleum hydrocarbon degradation pathways, including benzoate (KEGG map00362), toluene (KEGG map00623), and naphthalene degradation (KEGG map00626), under aerobic and oxygen-limited conditions.

**Figure S4.** Overview of cellular and metabolic pathways associated with microbial adaptation to oxygen-limited conditions, including glyoxylate metabolism (KEGG map00630) and nitrogen metabolism (KEGG map00910).
